# Association Between Organizational Culture and Emergency Medical Service Clinician Turnover

**DOI:** 10.3390/ijerph22050756

**Published:** 2025-05-12

**Authors:** Jacob C. Kamholz, Christopher B. Gage, Shea L. van den Bergh, Lakeshia T. Logan, Jonathan R. Powell, Ashish R. Panchal

**Affiliations:** 1 National Registry of Emergency Medical Technicians, Columbus, OH 43229, USA; gage.95@osu.edu (C.B.G.); sheab@nremt.org (S.L.v.d.B.); lakeshia.t.logan.mil@army.mil (L.T.L.); jpowell@imagetrend.com (J.R.P.); ashish.panchal@osumc.edu (A.R.P.); 2 Division of Health Services Management & Policy, College of Public Health, The Ohio State University, Columbus, OH 43210, USA; 3 Division of Epidemiology, College of Public Health, The Ohio State University, Columbus, OH 43210, USA; 4 Fort Sam Houston, The United States Army, San Antonio, TX 78234, USA; 5 ImageTrend, Eagan, MN 55121, USA; 6 Department of Emergency Medicine, The Ohio State University, Columbus, OH 43210, USA

**Keywords:** attrition, clinician retention, emergency medical services (EMS), healthcare workforce, job satisfaction, organizational behavior, organizational culture, prehospital care, turnover

## Abstract

The organizational culture significantly impacts employee behavior, satisfaction, and retention. Historically associated with hierarchical, fire-service-based structures, EMS cultures vary considerably, and their influence remains unclear. We aimed to identify organizational cultures within EMS agencies and examine their association with clinicians’ intent to leave within 12 months. We performed a cross-sectional survey of nationally certified civilian EMS clinicians aged 18–85 in the United States who recertified with the National Registry of Emergency Medical Technicians between October 2022 and April 2023. Respondents completed the validated Organizational Culture Assessment Instrument (OCAI), categorizing organizational cultures into adhocracy, clan, hierarchy, market, or mixed. Multivariable logistic regression evaluated the organizational culture and clinicians’ intention to leave, adjusting for personal demographics, workplace characteristics, and job satisfaction. In total, 30,762 survey respondents were included. Mixed and hierarchy cultures predominated, followed by clan, market, and adhocracy. Clinicians in adhocracy and hierarchy cultures had significantly higher adjusted odds of intending to leave EMS compared to clan and mixed. The organizational culture in EMS significantly influences clinicians’ intentions to leave. Adhocracy, hierarchy, and market cultures are associated with an increased likelihood of turnover, but clan-oriented environments do not increase risks. These findings suggest that optimizing EMS agency cultures towards collaboration and supportive environments may enhance clinician retention.

## 1. Introduction

Prehospital care is often delivered in high-risk and unstable environments that predispose EMS clinicians to significant daily and chronic stressors [1,2,3,4,5]. When these stressors are not effectively managed, they can lead to burnout and eventually leaving the EMS workforce [6,7,8,9,10]. Though many areas have reported high rates of clinician turnover, leading to workforce shortages, some EMS agencies also report low turnover rates and stable rosters [11]. This discrepancy raises the question: What factors help certain agencies retain their clinicians? One potential explanation lies in the EMS agency’s organizational culture and the support it confers to its clinicians.

The organizational culture comprises the shared beliefs, values, and assumptions guiding employee behavior and may play a pivotal role in shaping job satisfaction and retention [12]. When an organization’s culture aligns with the needs of its clinicians, it fosters enhanced job satisfaction and reduces turnover [7,9,13]. Research has highlighted that organizational cultures are largely shaped by agency leaders’ motivations and leadership styles, which, in turn, influence employees’ experiences [14,15,16,17,18,19,20]. Although EMS agencies have historically been thought to adopt hierarchical structures, reflecting their roots in the fire service, studies reveal a more nuanced landscape: only 25% of agencies have hierarchical cultures [13]. In comparison, mixed cultures predominate at more than 35% [13]. Despite these insights, little is known about how organizational culture influences EMS clinician retention.

Understanding this relationship is critical as it may offer actionable strategies for addressing workforce shortages. To better understand the impact of organizational culture, we aimed to evaluate the relationship between organizational culture and EMS clinicians’ leaving intentions. Specifically, our objective was to identify the prevailing cultures within EMS and evaluate their association with clinicians’ intent to leave the workforce in 12 months.

## 2. Materials and Methods

### 2.1. Study Design, Population, and Setting

We conducted a cross-sectional survey of current nationally certified civilian EMS clinicians aged 18–85 working in the United States who recertified with the National Registry of Emergency Medical Technicians from October 2022 to April 2023. This study method was selected to analyze EMS clinicians’ impressions of the organizational culture and leaving at one time point from a representative population. The National Registry of Emergency Medical Technicians (National Registry) is a nonprofit organization accredited by the National Commission for Certifying Agencies, responsible for certifying EMS clinicians in more than 46 states, territories, and federal agencies. At the end of their recertification process, all applicants were offered a one-time voluntary survey regarding their agency-related culture and the likelihood of leaving EMS in the next 12 months. This study was deemed exempt by the American Institutes for Research Institutional Review Board (project number: EX00621).

Nationally registered EMS clinician levels include emergency medical responder (EMR), emergency medical technician (EMT), advanced emergency medical technician (AEMT), and paramedic [1]. This study included only civilian EMTs, AEMTs, and paramedics aged 18–85 with at least one EMS job to represent the active prehospital patient care workforce most accurately. We excluded those who reported being primarily military EMS clinicians, as they follow a unique recertification process, and those who recertified at the emergency medical responder (EMR) level, because they rarely fill main prehospital EMS roles [21].

The recertification survey was administered via the Alchemer platform and had the potential to reach all recertification applicants for that particular cycle (over 150,000 eligible EMS clinicians annually) [22]. Each recertifying cohort represents a cross-section of the National EMS Certification database [21]. Since the survey was only accessible during recertification, a non-response survey was impossible in the current research paradigm. Response rates reflect this one-time survey’s administration. Following survey collection, its data were merged with those from the National EMS Certification database, which includes demographic and employment variables such as age, gender, certification level, EMS work experience, educational attainment, the number of EMS roles held, and full-time versus volunteer employment status.

### 2.2. Outcome

Our outcome of interest is the likelihood of leaving the EMS workforce within 12 months. Likelihood to leave the workforce was measured using a Likert scale in response to “How likely are you to leave your job?”: I definitely will leave, probably will leave, probably will not leave, and definitely will not leave. Answers were dichotomized into yes (definitely will or probably will leave) or no (probably will not leave or definitely will not). This outcome has been validated previously to assess the likelihood of leaving EMS in the next 12 months [7].

### 2.3. Exposure

To better understand the factors associated with EMS clinician attrition, we merged demographic and employment data from the National Registry Certification database with responses from the Organizational Culture Assessment Instrument [23,24,25]. This combined dataset enabled an in-depth analysis of the relationship between the organizational culture and clinicians’ intentions to leave EMS.

**Figure 1:**
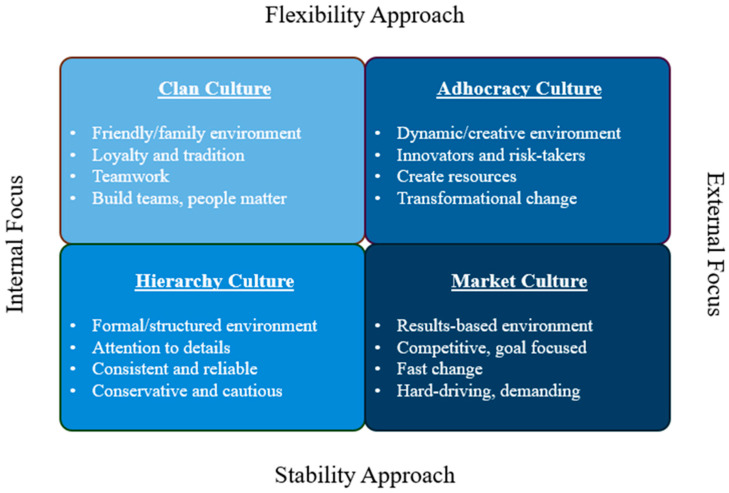
Organizational Culture Assessment Instrument (OCAI) and descriptions for the four major cultures: clan, hierarchy, adhocracy, and market. Each pair is arranged based on similarities in approach.

The organizational culture was assessed using the validated Organizational Culture Assessment Instrument (OCAI), grounded in the Competing Values Framework [23,25]. This framework evaluates organizational effectiveness based on two key dimensions: internal versus external focus and stability versus flexibility (Figure 1). These dimensions intersect to form a 2 × 2 matrix, defining four distinct organizational culture types. An internal focus emphasizes collaboration, development, and integration, whereas an external focus prioritizes market competition, customer satisfaction, and diversification. Stability-oriented organizations emphasize clear structures, detailed planning, and controlled environments. In contrast, flexibility-oriented organizations value adaptability and innovation, assuming that not everything can be predicted or controlled.

The OCAI survey evaluates six aspects of organizational culture: dominant characteristics, organizational leadership, management of employees, organization glue, strategic emphases, and criteria for success. Respondents rated each dimension on a scale from 1 (Poor) to 5 (Excellent), with scores reflecting their perception of the organization’s alignment with each cultural type. These responses were mapped onto the Competing Values Framework, categorizing organizational culture into one of four archetypes (Figure 1): Create Culture (Adhocracy), characterized by dynamism and entrepreneurship; Collaborate Culture (Clan), emphasizing people-oriented and team-driven environments; Control Culture (Hierarchy), focused on structured processes and efficiency; and Compete Culture (Market), driven by results and competition. The assigned culture was derived from scores within each dimension, and if the highest score was equal for two separate dimensions, the respondent was assigned a mixed culture. This comprehensive assessment enabled the categorization of EMS agencies based on their prevailing cultural characteristics, providing a framework to examine the relationship between organizational culture and clinician retention.

### 2.4. Measures

Demographic variables included age, binary sex, race/ethnicity, education level, and EMS certification level. Age was self-reported and categorized into six groups: <29, 29–37, 38–47, and >48 years. Race/ethnicity was self-identified using predefined categories: White, Black or African American, Asian, Hispanic or Latino, Native Hawaiian or Pacific Islander, and Other. Due to the relatively small proportion of minority EMS clinicians, race/ethnicity was dichotomized into non-minority (White, non-Hispanic) and minority (all other categories). Education level was categorized as high school diploma or GED or less, some college experience, associate degree, or bachelor’s degree and above. Individual certifications levels included EMT, AEMT, and paramedic.

Job satisfaction was assessed using the Spector job satisfaction survey, as previously validated and described [7,26,27,28]. Based on the JSS methodology, for respondents with missing data, conditional mean imputation was performed at the subscale level, replacing missing values with the individual’s mean response if at least two of the four subscale items were answered. This approach ensured the valid computation of total satisfaction scores while maintaining consistency with the JSS scoring methodology [27,29]. The total job satisfaction score was calculated as the sum of all 36 items (range: 36 to 216); scores of 36 to 108 were categorized as dissatisfied, 108 to 144 as ambivalent, and 144 to 216 as satisfied.

Organizational variables included agency and service types. Agency type was categorized into fire-based, hospital-based, governmental non-fire, private, or other organizations. Service type was classified according to the primary EMS services provided: 9-1-1 emergency response, medical (inter-facility) transport, combined 9-1-1 and medical transport, or other EMS services. These variables allowed for a nuanced analysis of how organizational models and service delivery approaches influence clinician retention.

### 2.5. Analysis

We calculated descriptive statistics to summarize the respondents’ key demographic, occupational, and agency characteristics. These statistics were presented as frequencies and percentages for categorical variables and as means with standard deviations (SD) or medians with interquartile ranges (IQRs) for continuous variables. Missing data were managed using mean conditional imputation, provided at least two of four questions within each organizational culture domain were completed; otherwise, responses were excluded.

Multivariable logistic regression was used to evaluate the association between our primary outcome (leaving EMS in 12 months) and primary exposure (organizational culture), adjusting for agency type, service type, full-time or volunteer status, number of EMS positions held, categorical age, certification level, race/ethnicity, and education level. Results are presented using odds ratios (ORs) and 95% confidence intervals (95% CIs). Model fit was assessed using the Hosmer–Lemeshow test, confirming its suitability for the observed data. Statistical significance was defined as a *p*-value of <0.05.

To manage potential sampling bias and ensure our estimates reflected the characteristics of the overall population, we conducted survey weighting based on the nationally certified EMS population demographics identified by the National Registry’s recertification dataset, as previously described [7]. Survey weights were calculated for demographic variables: age, sex, race, education, agency type, and number of EMS jobs. Weights were computed as the ratio of national to survey population proportions of each variable’s subgroups (e.g., education: high school/GED, some college, associates, etc.). Composite base weights were then assigned to every individual to account for the multiple variables/subgroups in the dataset. A sampling-weighted logistic regression analysis was conducted with the variables from the final model defined above. All statistical analyses were conducted using STATA software (Version 17, StataCorp, College Station, TX, USA) [30].

## 3. Results

**Figure 2:**
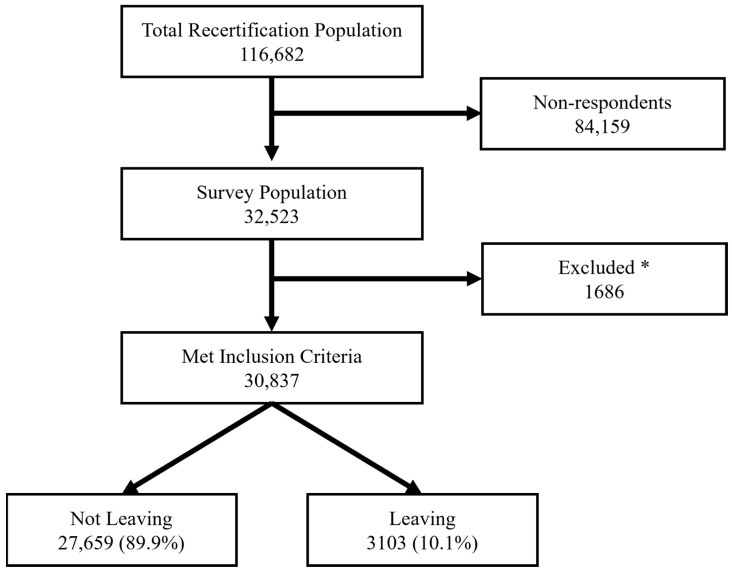
Flow chart of included population. * Individuals were excluded due to military roles or recertification at the emergency medical responder (EMR) level.

A total of 32,523 EMS clinicians completed the survey (Figure 2). The study population brought together a diverse range of EMS clinicians representing various age groups, certification levels, and agency types (Table 1). Similar to previous studies, this EMS clinician population was primarily male, white non-Hispanic, working in EMS full-time. The primary service type was 9-1-1 emergency response, and fire-based organizations dominated the responses, followed by private and hospital-based agencies. Among those completing the survey, mixed cultures were the most prevalent (36.9%), closely followed by hierarchy-oriented environments (25.1%). Clan-oriented organizations comprised 23.7% of the sample, with market (9.2%) and adhocracy (5.2%) cultures completing the distribution. A detailed demographic analysis of the population of respondents in each organizational culture is given in Appendix A.1.

In our adjusted multivariable logistic regression analysis, with mixed cultures as the referent, the relationship between organizational culture and clinicians’ intention to leave their agency within the next 12 months was examined. The results indicate that clinicians in adhocracy-oriented organizations (odds ratio [OR]: 1.31; 95% confidence interval [CI]: 1.10–1.55, *p* = 0.002) and hierarchy-oriented agencies (OR: 1.11; 95% CI: 1.00–1.23, *p* = 0.049) had significantly higher odds of planning to leave (Table 2). While the effect size of market had an association with higher odds of leaving, the *p*-value was not significant at the alpha < 0.05 level (OR: 1.14; 95% CI: 1.00–1.31, *p* = 0.055). In contrast, clinicians in clan-oriented organizations experienced no statistically significant difference in their likelihood to leave compared to those in mixed cultural settings (OR: 1.04; 95% CI: 0.92–1.16, *p* = 0.548).

To better reflect the national population, we give a survey weighted analysis in Appendix A.2. While the unweighted analysis did not find the market culture was significantly associated with leaving EMS in 12 months, the weighted analysis did (OR: 1.17; 95% CI: 1.02–1.34). There were no other differences compared to the unweighted sample in the directionality or magnitude.

## 4. Discussion

With a strong focus on maintaining the EMS workforce, we evaluated whether the type of EMS organizational culture may impact whether clinicians are likely to leave EMS in the next 12 months. Our analysis indicates that the organizational culture in EMS is associated with and influences clinician retention. Specifically, we noted that innovative/dynamic (Adhocracy) and formal/structured environments (Hierarchy) may be associated with an increased turnover risk. This was also noted in the competitive/results-based (Market) culture in the survey weighed analysis. Only a collaborative/friendly (Clan) environment does not appear to significantly increase attrition. This finding suggests that cultural dynamics that do not prioritize EMS clinicians may foster instability and unpredictability, contributing to turnover. These results underscore the importance of aligning the organizational culture with clinician retention strategies within EMS organizations.

These results are unique in that they evaluate the impact of an individual’s impression of their organizational culture on leaving intentions while adjusting for their job satisfaction. In previous work, job satisfaction has been noted as a strong driver of EMS leaving [7,26,31,32,33,34]. Among a survey of nationally certified EMS clinicians, job satisfaction was strongly associated with leaving EMS for both emergency medical technicians (OR 11.5; 95% CI 9.6–11.7) and paramedics (OR 13.5; 95% CI: 11.6–15.6) [7]. This was further substantiated using the validated Spector job satisfaction scale [26]. These results were consistent with other studies that described the intention of EMS clinicians to leave the profession [33,35,36]. Recognizing satisfaction as a strong driver, in this evaluation, when adjusting for job satisfaction, we still noted an association between organizational culture and leaving EMS. This supports the concept that leaving EMS is a multifactorial issue that needs to be addressed by improving the EMS agency’s organizational environment.

Instruments like the Organizational Culture Assessment Instrument (OCAI) could prove invaluable for EMS agencies aiming to identify and pinpoint opportunities for cultural improvement. From a practical perspective, our results suggest that refining the workplace culture is not merely an exercise; it is a strategic imperative for improving retention. EMS administrators should consider targeted interventions such as enhanced communication channels, regular team-building exercises, and leadership coaching that emphasizes both empathy and a clear organizational structure. These measures could help create an environment that supports clinician well-being while mitigating the pressures that drive turnover. 

We acknowledge several limitations in our work. First, the reliance on self-reported survey responses may have introduced biases. Second, our response rate was 27.9%, with 32,523 of 116,682 eligible EMS clinicians completing the survey. While this is a limitation, we compared demographic and professional characteristics of respondents and non-respondents using weighted and unweighted analyses (Appendix A.2), which revealed minimal differences in our primary outcome and main exposure, organizational culture. This supports the representativeness of our responding sample relative to the full recertifying population. Third, because our sample comprised clinicians recertifying with the National Registry of Emergency Medical Technicians, the findings may not be fully generalizable to EMS clinicians who certified solely through state processes. Additionally, while our findings demonstrate associations between the organizational culture and clinicians leaving EMS, the cross-sectional nature of this study prevents causal inference owing to temporality concerns. Finally, since we evaluated the perceptions of the organizational culture among individual EMS clinicians, further evaluation is required of agency-level cultural estimates and their association with leaving EMS.

## 5. Conclusions

EMS organizational cultures are associated with the influence of clinicians’ intent to leave the workforce. Organizations with a clan culture are not associated with leaving EMS, suggesting that agency types with a focus on friendly/family environments with high loyalty and teamwork are favored. These findings highlight the importance of evaluating and potentially optimizing the organizational culture within EMS agencies to mitigate clinician turnover.

## Figures and Tables

**Table 1:** Demographic and workforce characteristics of survey respondents by leaving status.

	Not Leaving n = 27,659 n (%)	Leaving n = 3103 n (%)	Total n = 30,762 n (%)
Age			
<29	6398 (23.1)	967 (31.2)	7365 (23.9)
29–37	7967 (28.8)	954 (30.7)	8921 (29.0)
38–47	6735 (24.4)	581 (18.7)	7316 (23.8)
48+	6559 (23.7)	601 (19.4)	7160 (23.3)
Sex			
Female	6914 (25.0)	951 (30.8)	7865 (25.6)
Male	20,692 (75.0)	2138 (69.2)	22,830 (74.4)
Missing	53	14	67
Race/ethnicity			
White, non-Hispanic	22,839 (83.8)	2431 (79.9)	25,270 (83.4)
Everyone else	4424 (16.2)	610 (20.1)	5034 (16.6)
Missing	396	62	458
Education level			
≤High school/GED	4095 (14.8)	321 (10.3)	4416 (14.4)
Some college	9220 (33.3)	963 (31.0)	10,183 (33.1)
Associates	5998 (21.7)	662 (21.3)	6660 (21.7)
Bachelor’s or above	8337 (30.2)	1156 (37.3)	9493 (30.9)
Missing	9	1	10
Certification level			
EMT	13,359 (48.3)	1417 (45.7)	14,776 (48.0)
AEMT	1769 (6.4)	170 (5.5)	1939 (6.3)
Paramedic	12,537 (45.3)	1516 (48.9)	14,047 (45.7)
Median years certified (IQR)	7 (4–13)	6 (4–12)	7 (4–13)
Full-time main EMS			
Full-time	21,438 (78.1)	2212 (71.6)	23,650 (77.4)
Missing	201	15	216
Volunteer main EMS			
Yes	3011 (10.9)	230 (7.4)	3241 (10.6)
Missing	78	9	87
EMS agency jobs			
1 job	20,002 (72.3)	2305 (74.3)	22,307 (72.5)
2 or more jobs	7657 (27.7)	798 (25.7)	8455 (27.5)
Agency type			
Hospital	3333 (12.1)	611 (19.8)	3944 (12.9)
Fire	12,961 (47.1)	812 (26.3)	13,773 (45.0)
Government non-fire	3209 (11.7)	392 (12.7)	3601 (11.8)
Private	5575 (20.3)	994 (32.2)	6569 (21.5)
Other	2433 (8.8)	277 (9.0)	2710 (8.9)
Missing	148	17	165
Service type			
9-1-1	19,243 (69.9)	1736 (56.3)	20,979 (68.5)
Medical transport	1431 (5.2)	277 (9.0)	1708 (5.6)
9-1-1 and medical transport	3856 (14.0)	559 (18.1)	4415 (14.4)
Other	2993 (10.9)	511 (16.6)	3504 (11.4)
Missing	136	20	156
NAEMSSO regions			
East	5963 (21.7)	687 (22.3)	6650 (21.8)
South	9605 (35.0)	1146 (37.1)	10,751 (35.2)
Great Lakes	4107 (15.0)	458 (14.8)	4565 (14.9)
Western Plains	4207 (15.3)	375 (12.1)	4582 (15.0)
West	3576 (13.0)	421 (13.6)	3997 (13.1)
Missing	201	16	217
Organizational culture			
Adhocracy	1389 (5.0)	208 (6.7)	1597 (5.2)
Clan	6673 (24.1)	607 (19.6)	7280 (23.7)
Hierarchy	6842 (24.7)	864 (27.8)	7706 (25.1)
Market	2411 (8.7)	422 (13.6)	2833 (9.2)
Mixed	10,344 (37.4)	1002 (32.3)	11,346 (36.9)

**Table 2:** Multivariable logistic regression of leaving EMS within the next 12 months by agency culture type. Adhocracy and hierarchy are associated with higher odds of leaving (alpha < 0.05).

Organizational Culture	Odds Ratio (95% CI)	p-Value
Mixed	Reference	
Adhocracy	1.31 (1.10–1.55)	0.002
Clan	1.04 (0.92–1.16)	0.548
Hierarchy	1.11 (1.00–1.23)	0.049
Market	1.14 (1.00–1.31)	0.055

## Data Availability

The data presented in this study are available on request from the corresponding author. Please note that the data are available only in English.

## Abbreviations

The following abbreviations are used in this manuscript:

AEMT	Advanced Emergency Medical Technician
CI	Confidence Interval
EMR	Emergency Medical Responder
EMS	Emergency Medical Services
EMT	Emergency Medical Technician
GED	General Educational Development
IQR	Interquartile Range
JSS	Job Satisfaction Survey
Medical Transport	Interfacility Transport
MIHCP	Mobile Integrated Health Community Paramedicine
NASEMSO	National Association of State EMS Officials
National Registry	National Registry of Emergency Medical Technicians
NR	National Registry
OCAI	Organizational Culture Assessment Instrument
OR	Odds Ratio
SD	Standard Deviation

## Appendix A

### Appendix A.1

**Table A1:** Demographic and workforce characteristics of survey respondents by organizational culture.

	Adhocracy n = 1600 n (%)	Clan n = 7293 n (%)	Hierarchy n = 7720 n (%)	Market n = 2842 n (%)	Mixed n = 11,382 n (%)
Age (years)					
<29	355 (22.2)	1689 (23.2)	1671 (21.6)	771 (27.1)	2888 (25.4)
29–37	460 (28.7)	2101 (28.8)	2164 (28.0)	821 (28.9)	3401 (29.9)
38–47	383 (23.9)	1716 (23.5)	1901 (24.6)	633 (22.3)	2698 (23.7)
48+	402 (25.1)	1787 (24.5)	1984 (25.7)	617 (21.7)	2395 (21.0)
Sex					
Female	417 (26.2)	2108 (29.0)	1911 (24.8)	805 (28.4)	2643 (23.3)
Male	1177 (73.8)	5173 (71.0)	5792 (75.2)	2030 (71.6)	8714 (76.7)
Missing	6	12	17	7	25
Race					
White, non-Hispanic	1320 (83.9)	6081 (84.6)	6380 (84.0)	2318 (83.1)	9226 (82.2)
Everyone else	254 (16.1)	1110 (15.4)	1214 (16.0)	472 (16.9)	2003 (17.8)
Missing	26	102	126	52	153
Certification level					
EMT	686 (42.9)	3735 (51.2)	3281 (42.5)	1297 (45.6)	5817 (51.1)
Advanced EMT	86 (5.4)	482 (6.6)	477 (6.2)	171 (6.0)	727 (6.4)
Paramedic	828 (51.7)	3076 (42.2)	3962 (51.3)	1374 (48.3)	4838 (42.5)
Education level					
≤High School/GED	220 (13.8)	1007 (13.8)	945 (12.2)	366 (12.9)	1887 (16.6)
Some college	548 (34.3)	2465 (33.8)	2510 (32.5)	918 (32.3)	3762 (33.1)
Associates	361 (22.6)	1539 (21.1)	1681 (21.8)	654 (23.0)	2440 (21.4)
Bachelor’s or above	470 (29.4)	2279 (31.3)	2581 (33.4)	903 (31.8)	3291 (28.9)
Missing	1	3	3	1	2
Years certified	8 (4–14)	6 (3–12)	7 (4–13)	7 (4–12)	7 (4–12)
Job status					
Part-time	368 (23.1)	1959 (27.1)	1489 (19.4)	586 (20.8)	2518 (22.3)
Full-time	1224 (76.9)	5271 (72.9)	6186 (80.6)	2234 (79.2)	8786 (77.7)
Missing	8	63	45	22	78
Main EMS job volunteer					
No	1418 (89.0)	6189 (85.1)	7146 (92.8)	2618 (92.4)	10,125 (89.2)
Yes	176 (11.0)	1082 (14.9)	558 (7.2)	216 (7.6)	1221 (10.8)
Missing	6	22	16	8	36
Number of EMS jobs					
1 job	1125 (70.3)	5286 (72.5)	5604 (72.6)	2022 (71.1)	8325 (73.1)
2 or more jobs	475 (29.7)	2007 (27.5)	2116 (27.4)	820 (28.9)	3057 (26.9)
Agency type					
Hospital	225 (14.1)	902 (12.4)	1020 (13.3)	408 (14.4)	1398 (12.4)
Fire	612 (38.4)	3251 (44.8)	3265 (42.5)	1081 (38.2)	5594 (49.4)
Government non-fire	235 (14.7)	843 (11.6)	1083 (14.1)	305 (10.8)	1143 (10.1)
Private	368 (23.1)	1531 (21.1)	1744 (22.7)	734 (26.0)	2209 (19.5)
Air medical	67 (4.2)	249 (3.4)	219 (2.8)	144 (5.1)	372 (3.3)
Other	88 (5.5)	473 (6.5)	357 (4.6)	155 (5.5)	597 (5.3)
Missing	5	44	32	15	69
Service type					
9–1-1	1059 (66.7)	5076 (69.9)	5307 (69.0)	1755 (62.0)	7829 (69.2)
Medical transport	89 (5.6)	344 (4.7)	407 (5.3)	257 (9.1)	614 (5.4)
9-1-1 and medical transport	253 (15.9)	1061 (14.6)	1127 (14.7)	443 (15.6)	1542 (13.6)
Clinical services	83 (5.2)	393 (5.4)	456 (5.9)	197 (7.0)	669 (5.9)
MIHCP	24 (1.5)	36 (0.5)	36 (0.5)	22 (0.8)	99 (0.9)
Other	79 (5.0)	348 (4.8)	353 (4.6)	158 (5.6)	562 (5.0)
Missing	13	35	34	10	67
NASEMSO region					
East	324 (20.4)	1556 (21.5)	1719 (22.4)	612 (21.7)	2454 (21.7)
South	592 (37.2)	2264 (31.3)	2743 (35.7)	1013 (35.9)	4161 (36.8)
Great Lakes	260 (16.3)	1168 (16.1)	1147 (14.9)	434 (15.4)	1568 (13.9)
Western Plains	245 (15.4)	1286 (17.8)	1081 (14.1)	372 (13.2)	1605 (14.2)
West	170 (10.7)	966 (13.3)	985 (12.8)	387 (13.7)	1507 (13.3)
Missing	9	53	45	24	87

### Appendix A.2

**Table A2:** Odds ratios (ORs) and 95% confidence intervals (CIs) comparing unweighted and weighted multivariable logistic regression models of EMS clinicians’ likelihood of leaving within 12 months. Weighted models incorporate survey weights to represent the full population, whereas unweighted models use only the sample.

Organizational	Weighted	Unweighted
Cultures		
Adhocracy	1.29 (1.09–1.54) *	1.31 (1.10–1.55) *
Clan	1.05 (0.93–1.18)	1.04 (0.92–1.16)
Hierarchy	1.12 (1.01–1.25) *	1.11 (1.00–1.23) *
Market	1.17 (1.02–1.34) *	1.14 (1.00–1.30)

Significance (*) at the alpha < 0.05 level.

## References

[1] National Association of EMS State Officials National EMS Scope of Practice Model 2019 (Report No. DOT HS 812-666). https://www.nhtsa.gov/node/103856.

[2] Maguire B.J., Smith S. (2013). Injuries and fatalities among emergency medical technicians and paramedics in the United States. Prehospital Disaster Med..

[3] (1996). EMS Agenda for the Future.

[4] Amro T.M., Arcos Gonzalez P., Montero Vinuales E., Castro Delgado R. (2022). Impact of COVID-19 Pandemic on Stress and Burnout Levels amongst Emergency Medical Technicians: A Cross-Sectional Study in Spain. Ann. Med..

[5] Srikanth P., Monsey L.M., Meischke H.W., Baker M.G. (2022). Determinants of Stress, Depression, Quality of Life, and Intent to Leave in Washington State Emergency Medical Technicians During COVID-19. J. Occup. Environ. Med..

[6] Powell J.R., Gage C.B., Crowe R.P., Rush L.J., MacEwan S.R., Dixon G., McAlearney A.S., Panchal A.R. (2025). National Evaluation of Emergency Medical Services Clinician Burnout and Workforce-Reducing Factors. JACEP Open.

[7] Gage C.B., Cooke C.B., Powell J.R., Kamholz J.C., Kurth J.D., van den Bergh S., Panchal A.R. (2024). Factors Associated With Emergency Medical Clinicians Leaving EMS. Prehospital Emerg. Care.

[8] Blau G., Chapman S.A. (2016). Why do Emergency Medical Services (EMS) Professionals Leave EMS?. Prehospital Disaster Med..

[9] Lu D.W., Shin J., Wan C., Rea T.D., Crowe R.P., Meischke H.W., Counts C.R. (2023). Burnout and Workplace Incivility Among Emergency Medical Services Practitioners: A Preliminary Report. Prehospital Emerg. Care.

[10] Kaplan G.R., Frith T., Hubble M.W. (2024). Quantifying the prevalence and predictors of burnout in emergency medical services personnel. Ir. J. Med. Sci..

[11] American Ambulance Association 2024 EMS Employee Turnover Study. https://ambulance.org/sp_product/2024-turnover.

[12] Brimhall K.C., Lizano E.L., Mor Barak M.E. (2014). The mediating role of inclusion: A longitudinal study of the effects of leader–member exchange and diversity climate on job satisfaction and intention to leave among child welfare workers. Child. Youth Serv. Rev..

[13] Cash R.E., White-Mills K., Crowe R.P., Rivard M.K., Panchal A.R. (2019). Workplace Incivility Among Nationally Certified EMS Professionals and Associations with Workforce-Reducing Factors and Organizational Culture. Prehospital Emerg. Care.

[14] Tsai Y. (2011). Relationship between organizational culture, leadership behavior and job satisfaction. BMC Health Serv. Res..

[15] Bradley E.H., Brewster A.L., McNatt Z., Linnander E.L., Cherlin E., Fosburgh H., Ting H.H., Curry L.A. (2018). How guiding coalitions promote positive culture change in hospitals: A longitudinal mixed methods interventional study. BMJ Qual. Saf..

[16] Curry L.A., Brault M.A., Linnander E.L., McNatt Z., Brewster A.L., Cherlin E., Flieger S.P., Ting H.H., Bradley E.H. (2018). Influencing organisational culture to improve hospital performance in care of patients with acute myocardial infarction: A mixed-methods intervention study. BMJ Qual. Saf..

[17] Scott T., Mannion R., Marshall M., Davies H. (2003). Does organisational culture influence health care performance? A review of the evidence. J. Health Serv. Res. Policy.

[18] Goodson H. (2016). Establishing Identity: A New Zealand perspective on organizational culture in EMS. JEMS.

[19] Burns K.E.A., Pattani R., Lorens E., Straus S.E., Hawker G.A. (2021). The impact of organizational culture on professional fulfillment and burnout in an academic department of medicine. PLoS ONE.

[20] Tate K., Penconek T., Dias B.M., Cummings G.G., Bernardes A. (2023). Authentic leadership, organizational culture and the effects of hospital quality management practices on quality of care and patient satisfaction. J. Adv. Nurs..

[21] Rivard M.K., Cash R.E., Mercer C.B., Chrzan K., Panchal A.R. (2021). Demography of the National Emergency Medical Services Workforce: A Description of Those Providing Patient Care in the Prehospital Setting. Prehospital Emerg. Care.

[22] Alchemer, LLC. https://www.alchemer.com/.

[23] Cameron K., Quinn R. (2011). Diagnosing and Changing Organizational Culture: Based on the Competing Values Framework.

[24] Heritage B., Pollock C., Roberts L. (2014). Validation of the organizational culture assessment instrument. PLoS ONE.

[25] Lamers M., Bremer M. OCAI Online. https://www.ocai-online.com/about-the-Organizational-Culture-Assessment-Instrument-OCAI.

[26] Gage C.B., Logan L., Kamholz J.C., Powell J.R., van den Bergh S.L., Kenah E., Panchal A.R. (2025). The Spector Job Satisfaction Survey: Associations of Satisfaction with Leaving EMS. Prehospital Emerg. Care.

[27] Spector P.E. (1985). Measurement of human service staff satisfaction: Development of the Job Satisfaction Survey. Am. J. Community Psychol..

[28] Thielmann B., Schwarze R., Böckelmann I. (2023). A Systematic Review of Associations and Predictors for Job Satisfaction and Work Engagement in Prehospital Emergency Medical Services-Challenges for the Future. Int. J. Environ. Res. Public Health.

[29] Spector P.E. Paul Spector Industrial and Organizational Psychology. https://paulspector.com/assessments/pauls-no-cost-assessments/job-satisfaction-survey-jss/.

[30] Stata. https://www.stata.com/.

[31] Blau G., Chapman S. (2011). Retrospectively exploring the importance of items in the decision to leave the emergency medical services (EMS) profession and their relationships to life satisfaction after leaving EMS and likelihood of returning to EMS. J. Allied Health.

[32] Thielmann B., Schnell J., Bockelmann I., Schumann H. (2022). Analysis of Work Related Factors, Behavior, Well-Being Outcome, and Job Satisfaction of Workers of Emergency Medical Service: A Systematic Review. Int. J. Environ. Res. Public Health.

[33] Patterson P.D., Moore C.G., Sanddal N.D., Wingrove G., LaCroix B. (2009). Characterizing job satisfaction and intent to leave among nationally registered emergency medical technicians: An analysis of the 2005 LEADS survey. J. Allied Health.

[34] Stefurak T., Morgan R., Johnson R.B. (2020). The relationship of public service motivation to job satisfaction and job performance of emergency medical services professionals. Public Pers. Manag..

[35] Rivard M.K., Cash R.E., Woodyard K.C., Crowe R.P., Panchal A.R. (2020). Intentions and Motivations for Exiting the Emergency Medical Services Profession Differ Between Emergency Medical Technicians and Paramedics. J. Allied Health.

[36] Cash R.E., Crowe R.P., Agarwal R., Rodriguez S.A., Panchal A.R. (2018). Exiting the Emergency Medical Services Profession and Characteristics Associated with Intent to Return to Practice. Prehospital Emerg. Care.

